# An Inducible Expression System to Measure Rhodopsin Transport in Transgenic *Xenopus* Rod Outer Segments

**DOI:** 10.1371/journal.pone.0082629

**Published:** 2013-12-06

**Authors:** Xinming Zhuo, Mohammad Haeri, Eduardo Solessio, Barry E. Knox

**Affiliations:** Departments of Neuroscience and Physiology, Biochemistry and Molecular Biology and Ophthalmology, SUNY Upstate Medical University, Syracuse, New York, United States of America; Muséum National d'Histoire Naturelle, France

## Abstract

We developed an inducible transgene expression system in *Xenopus* rod photoreceptors. Using a transgene containing mCherry fused to the carboxyl terminus of rhodopsin (Rho-mCherry), we characterized the displacement of rhodopsin (Rho) from the base to the tip of rod outer segment (OS) membranes. Quantitative confocal imaging of live rods showed very tight regulation of Rho-mCherry expression, with undetectable expression in the absence of dexamethasone (Dex) and an average of 16.5 µM of Rho-mCherry peak concentration after induction for several days (equivalent to >150-fold increase). Using repetitive inductions, we found the axial rate of disk displacement to be 1.0 µm/day for tadpoles at 20 °C in a 12 h dark /12 h light lighting cycle. The average distance to peak following Dex addition was 3.2 µm, which is equivalent to ~3 days. Rods treated for longer times showed more variable expression patterns, with most showing a reduction in Rho-mCherry concentration after 3 days. Using a simple model, we find that stochastic variation in transgene expression can account for the shape of the induction response.

## Introduction

*Xenopus* photoreceptors have played an important role in understanding the cell biology of membrane assembly [1-4], retinal disease [5-7] and ciliary transport [8]. A major advantage is that *Xenopus* has much larger photoreceptors than mammals, which make them an exquisite system for high-resolution microscopic imaging [5,9-11]. The photoreceptors develop rapidly, forming light-sensitive outer segment (OS) membranes within a week post-fertilization [12,13] and expression of fluorescently tagged proteins are readily expressed [14]. The OS contains high concentrations (~3 mM) of the integral membrane protein rhodopsin [15], which is synthesized in the inner segment and then rapidly delivered to the base of the OS for incorporation into disk membranes [16-20]. Rhodopsin is free to move laterally within a disk membrane [21], however there is no axial movement between disks. OS renewal is accomplished as membrane disks are displaced progressively outwards, with eventual phagocytosis by the retinal pigment epithelium [16-19,22,23]. Therefore, the distance along the OS axis from the base is linearly related to the time elapsed since incorporation for 4-6 weeks and thus offers an opportune model to investigate membrane protein synthesis and processing. 

**Figure 1:**
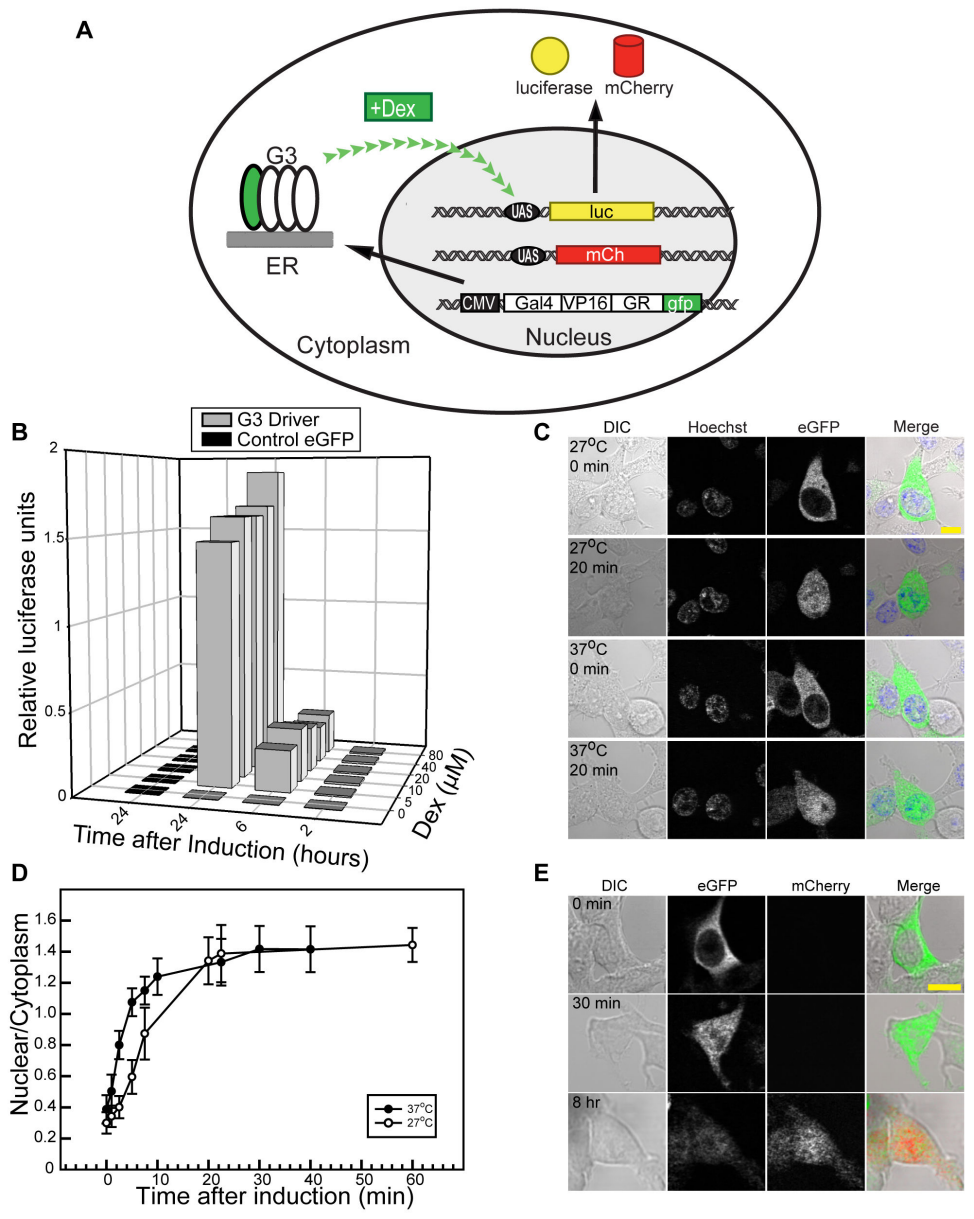
G3U inducible system in mammalian cell culture. (A) Diagram of G3U system using luciferase (pCMV:G3 and pUAS:Luciferase) or mCherry reporters (top, pCMV:G3 and pUAS:mCherry). A CMV promoter drives transcription of a chimeric transcription factor, G3, which encodes contains a GAL4 DNA binding domain, the VP16 transcription activation domain, a rat glucocorticoid receptor binding domain (GR) and eGFP. Synthesized G3 protein localizes to the cytosol. Dex treatment triggers the dimerization of G3, which translocates into the nucleus. Nuclear G3 activates a second construct containing five tandem repeats of the UAS sequence upstream of the hsp70 minimal promoter. The reporter gene (luciferase or mCherry) is under the control of this system. (B) Luciferase assay of G3U inducible system in cell culture. HEK293T cells transfected with pCMV:G3 and pUAS:Luciferase were lysed and luciferase activity measured at different concentrations (0-80 µM) and treatment durations (2-24 hr) of Dex. Relative luciferase activity is plotted as a function of duration and Dex concentration. (C) Live cell imaging of G3 translocation after induction. HEK293 cells were transfected with pCMV:G3 and pUAS:mCherry and induced with 10 μM Dex at 27°C and 37°C. Confocal images were taken before and 20 min after induction; G3 (eGFP), nucleus (Hoechst). Scale bar is 10 μm. (D) Nuclear translocation rate of G3 in HEK293T cells at different temperatures. Fluorescence intensity was measure in nuclear and cytoplasm of live 293T cells at 27°C and 37 °C. Dex (10 μM) was added and mixed into medium. Error bars represent standard deviation (n = 17 at 37 °C and n = 15 at 27 °C). (E) mCherry reporter expression after induction. HEK293T cells transfected with pCMV:G3-GFP and pUAS:mCherry were induced with 10 μM Dex and fixed at different times after induction. G3 (eGFP) and mCherry images show the movement of G3 and expression of mCherry. Scale bar is 10 μm.

**Figure 2:**
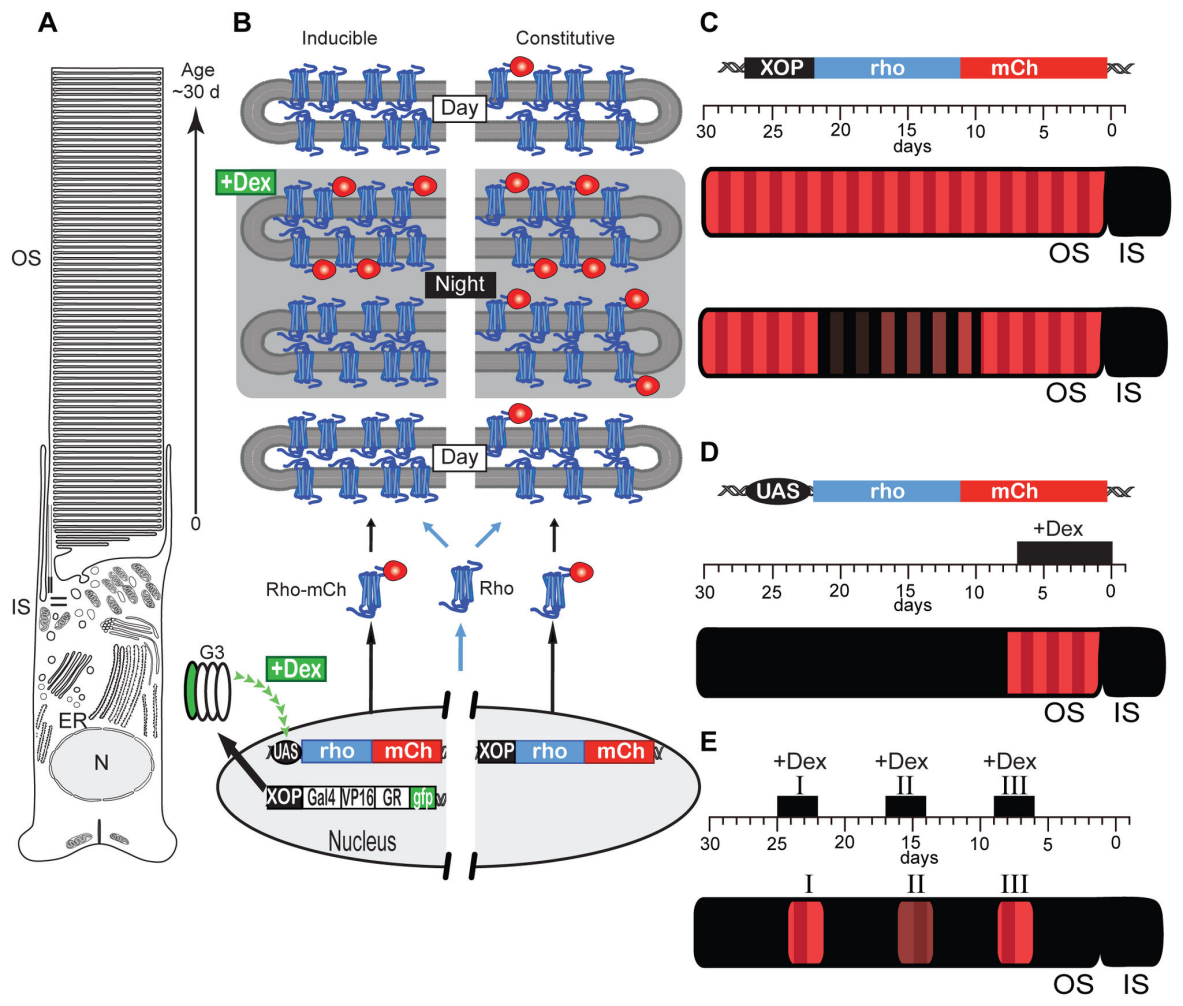
An inducible expression system for *Xenopus* rods. (A) Schematic diagram of a *Xenopus* rod. In *Xenopus*, there is a daily synthesis of approximately 80 discs, and the previous disks are displaced apically. Thus, the distance of disks from the base of the OS is linearly related to the time after induction. (B) Schematic diagram of a XOP:G3U-Rho-mCherry system (left) and a XOP:Rho-mCherry constitutive expression system (right). Rod-specific expression is accomplished using the *Xenopus* rhodopsin promoter (XOP) driving transcription of G3. Dex treatment of animals transgenic for both XOP:G3 and pUAS:Rho-mCherry induces synthesis of Rho-mCherry that is transported and integrated into the rod outer segment (OS) disk membranes. Rods with XOP:Rho-mCherry express the Rho-mCherry constitutively. (C) Constitutive expression of XOP:Rho-mCherry transgene (top). There are two kinds axial variation of Rho-mCherrry expression in the OS: diurnal variation (middle) and long-term variation (lower). (D) Dex induction treatment paradigm 1. Tadpoles (St. 54) were treated with 10 µM Dex for 7 days and then sacrificed immediately before imaging. (E) Dex induction treatment paradigm II. Tadpoles (St. 54) received repetitive 3-day 10 µM Dex inductions (black boxes), each followed by a 5 day interval without Dex. Seven days after the last induction, retinas were explanted immediately before imaging.

In order to more precisely control expression of the transgene in *Xenopus* rods, an inducible expression system is invaluable. There have been three inducible systems developed in *Xenopus* based upon the Gal4-UAS [24,25], Tet-On [26] and heat shock [27-29] strategies. The Gal4-UAS systems (Gal4 DNA binding domain was fused with a ligand binding domain from progesterone [24] or glucocorticoid [25] hormone receptors) respond well to low concentration of inducer during *Xenopus* development. However, they have significant leakiness in the absence of inducer and pleiotropic variation. The Tet-On inducible system was successfully employed in *Xenopus* to study thyroid hormone response gene expression [26]. However, rtTa binds to tetO weakly even without doxycycline, which leads to a basal level expression of reporter genes [30,31]. Heat-shock promoters have been used to study Wnt signalling [27,28] and HNF1 related organogenesis [29]. A big advantage of this system is that only a short-term heat shock is needed to turn on the system and no inducer is needed. However, the heat-shock promoter systems are not suitable for long-term treatment. To overcome these limitations, we designed a system that would have tight control of expression and provide reproducible induction responses (Figures 1A and 2A). This system is based upon a GAL4-VP16 transcriptional activator fused to a fragment of the glucocorticoid receptor, which makes nuclear localization dexamethasone (Dex)-sensitive [32]. An eGFP protein is fused to the C-terminus of GAL4-VP16-GR chimeric protein to allow detection of transcriptional activator translocation. This system was placed under control of the *Xenopus* rhodopsin promoter (XOP) to direct expression specifically in rods. To follow rhodopsin transport, a Rho-mCherry fusion protein was placed under the control of a UAS-hsp promoter. Thus, Dex addition triggers GAL4-VP16-GR-eGFP (G3) transport into the nucleus and initiates the transcription of the Rho-mCherry reporter. We used high-resolution confocal microscopy of single rods treated with Dex to characterize spatial and temporal characteristics of induction responses and displacement rates of rhodopsin in the OS. 

## Materials and Methods

### Expression constructs

Fusion constructs were spliced by overhang extension PCR primers (IDT, Coralville, IA) with Cloned Pfu (Stratagene, La Jolla, CA). Point mutations and small deletions or insertions were generated using QuickChange methods with Turbo Pfu (Stratagene, La Jolla, CA). All constructs were confirmed by DNA sequencing. The Gal4-VP16-GR module (consisting of 147 amino acids of *S. cerevisiae* Gal4 N-terminal transactivation domain, 59 amino acids of herpes viral protein VP16 and 266 amino acids of rat glucocorticoid receptor protein C terminal domain) was amplified from TOPtk-iGFP plasmid [25] and inserted into peGFP-N1 vector (Clonetech, Mountain View, CA) with poly-Gly linker (LEPLEGTGGGGG) to create the pCMV:G3 plasmid. The CMV promoter was replaced with XOP (-503/+41) promoter [33] to create the XOP:G3 construct. A fragment containing five copies of UAS immediately upstream of Hsp promoter was amplified from pUAS:GFP [34] and subcloned into pGL2 (Promega, Madison, WI) to create pUASLuc, pmCherry-N1 (Clonetech, Mountain View, CA) to create pUAS:mCherry and replacing XOP promoter in pRho-mCherry [5] to create pUAS:Rho-mCherry. All plasmids were linearized with Nhe I (NEB, Ipswich, MA) prior to transgenesis.

### Mammalian Cell Culture and Luciferase Reporter Assay

HEK293T cells (ATCC, Manassas, VA) were cultured in DMEM with 10% FBS and 1 mM L-Glutamine. Cells were seeded at 75,000 cells/ml one day before transfection. Cell transfections were performed using a total of 1 µg of DNA and 3 µl Fugene 6 (Roche, Branchburg, NJ) in 100 µl DMEM for 2 ml of culture. Cells were transfected with pCMV:G3 and either pUAS:Luc or pUAS:mCherry constructs. Empty pCS2 (D. Turner, University of Michigan) was included in the transfection medium to bring the total DNA to 1 µg. Cells were harvested 48 h post-transfection and luciferase activity was determined with Bright-Glo Luciferase Assay System (Promega, Madison, WI) using a Synergy 2 Multi-Mode Microplate Reader (BioTek, Winooski, VT) according to the manufacturer's instructions.

### Transgenesis and induction procedure

Transgenic *Xenopus laevis* were generated by restriction enzyme mediated integration [35-37]. Tadpoles were raised in a 12 h dark/12 h light cycle at 20 °C. During induction, tadpoles had daily water changes and replenished with fresh 10 µM Dex (Sigma-Aldrich). All animal handling and experiments were in agreement with the animal care and use guidelines of the Association for Research in Vision and Ophthalmology (ARVO). This study was done under the approval of the SUNY Upstate Medical University Committee on the Human Use of Animals (CHUA No. 209).

### Confocal microscopy image acquisition setting

Cells were imaged with a confocal microscope (LSM510 META; Carl Zeiss, Germany) using LSM acquisition software (Carl Zeiss, Germany). Images were acquired with a Plan-Apochromat 63× oil immersion objective (NA 1.4). The pinhole was adjusted to obtain 1.24 Airy units for the fluorophore of shortest wavelength excitation/emission properties. mCherry fluorescence was detected by using an HeNe1 laser (excitation at 543nm, power 20–60%), a main dichroic beam splitter (MBS) HFT UV/488/543/633-nm followed with a dichroic beam splitter (DBS) NFT490-nm for excitation, and a 650/710-nm band pass filter. GFP fluorescence was detected using an Argon laser with an excitation line at 488 nm (power 0.5–2%), a MBS HFT UV/488/543/633-nm follow with a DBS NFT545-nm for excitation, and a 500/530-nm band pass emission filter. Hoechst 33342 staining was detected using a two-photon Chameleon laser with an excitation at 800 nm (power 4-8%), a MBS HFT KP650-nm follow with a DBS NFT490-nm for excitation, and a 435/485-nm band pass emission filter. For dual-colour acquisition, images were sequentially acquired in line scan mode (average line = 2). 

### Immunohistochemistry

Dark-adapted tadpoles were fixed with 4% paraformaldehyde in PBS overnight and processed for cryostat section and immunostaining as previously described [5]. 

### Live Rod imaging

*Xenopus* were dark adapted for at least five hours before being euthanized for the experiment. The retina was isolated and cut into small pieces in oxygenated Ringer’s solution, (111 mM NaCl, 2 mM KCl, 1 mM CaCl_2_, 1 mM MgCl_2_, 0.5 mM MgSO_4_, 0.5 mM NaH_2_PO_4_, 3 mM HEPES, 10 mM glucose, 0.01 mM EDTA, pH 7.6). Portions of retina were loaded into a glass chamber (No. 1 coverslip affixed to the bottom of a Fisher 3 cm petri dish with a 3 mm milled hole) and then sealed with a No.1 coverslip [9]. A dim red light was used for all steps of tissue manipulation. 

### Calibration of fluorescence protein concentration with confocal microscope

Purified mCherry concentration was determined with a UV-visible spectrophotometer (Beckman, Brea CA) using the extinction coefficient 72 x 10^3^ M^-1^cm^-1^ at λ=587 nm [38]. The mCherry protein stock was diluted at various concentrations with Tris-HCl (pH 7.8-8.8) and then loaded into a 15 µl chamber. Fluorescence intensity was measured using the LSM confocal microscope with the same optical settings as described in live rod imaging. The mCherry measurements were obtained 10 µm away from the surface of the cover glass. The mCherry solution was imaged with the HeNe1(543 nm) laser at various power and gain values. The measured fluorescence intensity was plotted versus concentration at different laser power levels. We then used these plots to calibrate mCherry concentration from fluorescence intensity in images of live rods. 

### Analysis of confocal image data

Images from live rods were analysed with Image J software (NIH). Central axial z-sections of rods were extracted from stacks. Heat-maps of rods were plotted with "Heatmap from stacks" plug-in for Image J (http://www.samuelpean.com/heatmap-from-stack). Fluorescence intensity of each rod along their axis were measured, constrained normalized with the maximum intensity set to 100% and plotted against the distance from IS/OS junction. The average Rho-mCherry fluorescence intensity distribution in OS from multiple rods was calculated as follows. First, the position corresponding to the 50% maximal intensity in the rising phase of each induction response in each rod was set to 0 µm. The rods were then aligned at this reference position. The fluorescent intensities from different induction responses were then averaged and constrained normalized. All intensity profiles, dot plots and bar graphs were generated with Sigma Plot 11.0 (Systat Software, Inc., Chicago, IL). The statistical analysis was done using Student’s t-test in Sigma Plot 11.0 (Systat Software). Gaussian, exponential and sinusoidal curve fitting also used corresponding global fitting function in Sigma Plot 11.0 (Systat Software). 

## Results

### Dexamethasone induced expression: Cell culture

To determine the efficiency of regulation achievable by the G3U inducible system, we first utilized transfected HEK293 cells to measure the magnitude of induction and the leakiness of the UAS promoter. Cells were transfected with pUAS:Luc and either pCMV:eGFP (control) or pCMV:G3 for luciferase assay. Different concentrations of Dex (0-100 μM) were added into the medium and incubated for various periods of time (0-24 h) prior measurement of luciferase activity (Figure 1B). In the absence of Dex, cells transfected with either pCMV:eGFP (control) or pCMV:G3 had no significant luciferase activity compared to untransfected cells. We observed robust responses (~150-fold induction) in cells transfected with pCMV:G3 when Dex was included in the culture media, even at the lowest concentrations tested (5 μM). We were able to detect expression of luciferase within 6 hours of Dex addition, and the expression steadily increased after that time. Thus, the G3U system exhibits tight control of expression and rapid induction in HEK293 cells. To study G3U translocation using confocal microscopy, we induced cells transfected with pCMV:G3 and pUAS:mCherry with 10 μM Dex and observed the fluorescence distribution pattern over time (Figure 1C and 1D). The eGFP nuclear/cytoplasm intensity ratio equilibrated within 20 min of Dex addition, with a half time to reach a steady distribution was ~3 min at 37 °C, in close agreement to previously reported values of a GFP-GR protein [39]. Since we intend to utilize this system in transgenic *Xenopus*, which are housed at lower temperatures, we also measured the rate at 27 °C (Figure 1C and 1D) and found that it is 2.2-fold slower than at 37 °C. We also tested Rho-mCherry, which is suitable for measuring rhodopsin transport in transgenic *Xenopus* rods [5], for induction response in transfected cells. We first observed Rho-mCherry fluorescence in cells after 4 h of treatment (*data not shown*) with steady increases after 4 h (Figure 1E). Thus, these preliminary experiments suggested that G3U system functions as designed in mammalian cells and thus be studied in transgenic *Xenopus*. 

### Dexamethasone Induced Expression: Transgenic Rods

We generated transgenic *Xenopus* tadpoles, iXRC, with two separately integrated transgenes: XOP:G3 and pUAS:Rho-mCherry (Figure 2B). Rod-specific expression is accomplished using the *Xenopus* rhodopsin promoter (XOP) driving transcription of G3. Dex treatment of iXRC animals induces synthesis of Rho-mCherry that is transported to the rod outer segment (OS) disk membranes. The fluorescent disks are moving outward continually. Thus, the distance of disks from the base of the OS correlates to the time after induction (Figure 2B). All animals exhibited Dex-dependent expression of Rho-mCherry and one male founder, iXRC1, was chosen for expansion and F1 animals were subjected to detailed imaging analysis. We produced tadpoles (Stage 54-58) from the iXRC1 founder and treated them with various concentrations of dexamethasone (0 to 500 µM) for seven days, during which time we did not detect any significant adverse effects on tadpole health (*data not shown*). Dexamethasone treatment had no obvious effect on organization of the retina or cell number, and the OS appeared normal (*data not shown*). 

**Figure 3:**
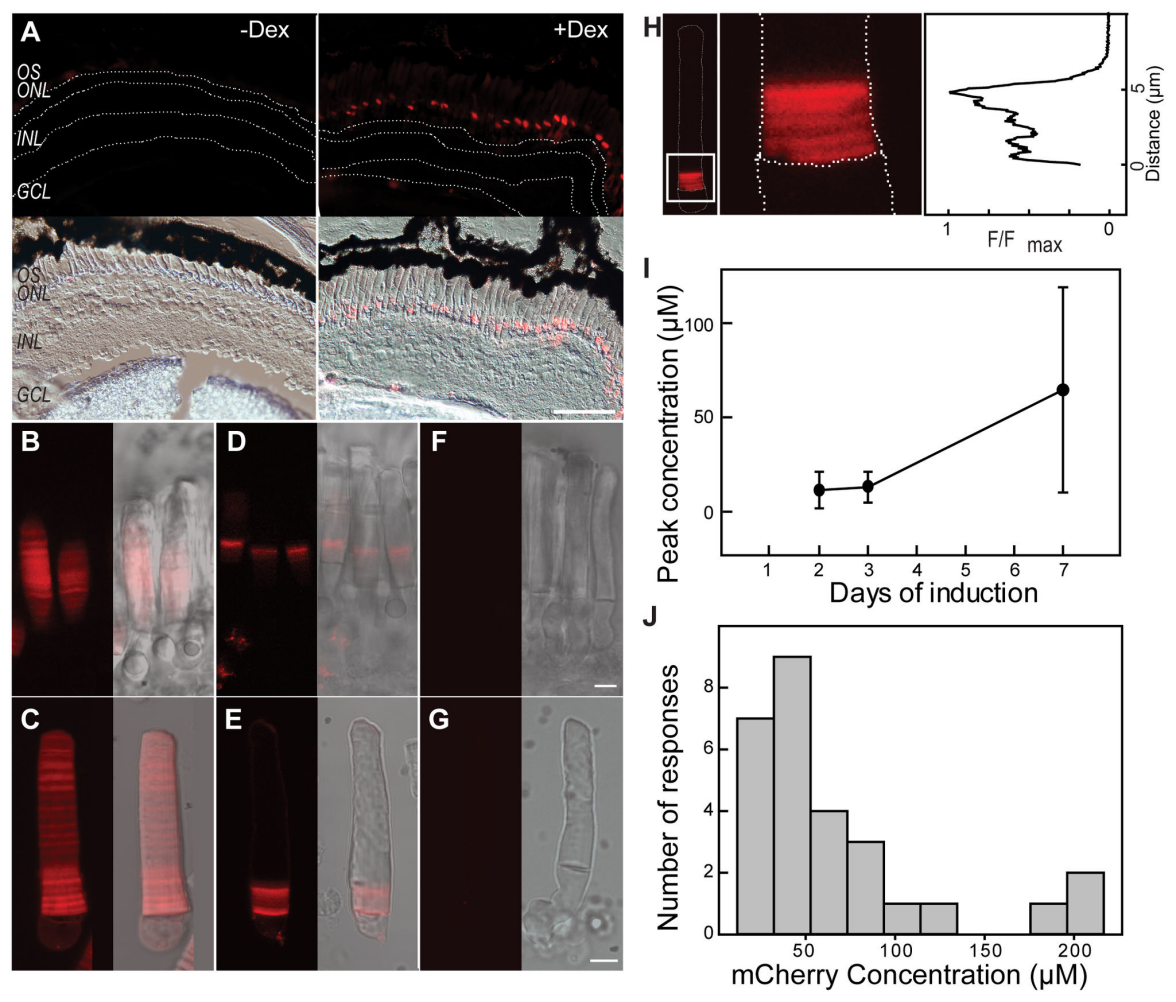
Induction responses of G3U system in *Xenopus* rods. (A) Micrographs of retinas from iXRC1 tadpoles transgenic for both XOP:G3 and pUAS:Rho-mCherry (G3U^+^). Tadpoles (St. 52-56) were treated (right) or untreated (left) with Dex for three days. Three days later, retinas were fixed and processed for fluorescence (top) or DIC (bottom) microscopy. Fluorescence was merged with DIC for reference. Retinal layers are indicated as follow: OS, rod outer segment; ONL, outer nuclear layer; INL, inner nuclear layer; GCL, ganglion cell layer. Scale bar is 50 μm. (B-G) Live rod imaging. Tadpoles (St. 52-56) were treated with 10 μM Dex for seven days, dissected under dim red light and imaged using confocal microscopy (left) and merged with DIC for reference (right). (B), (C) Rods in a retinal chip and single rod from tadpoles constitutively expressing Rho-mCherry. (D), (E) Rods of retinal chip and single rod from tadpoles transgenic for XOP:G3 and pUAS:Rho-mCherry treated with Dex for 7 days prior to imaging. (F), (G) Rods of retinas chip and single rod from wild type tadpoles. Scale bar represents 5 μm. (H) Diurnal banding in rods treated with Dex for seven days. Fluorescent micrograph of the rod (left) with the cell body outlined, with enlarged image of the IS/OS junction (middle) and relative fluorescence intensity along the axis (right). (I) Peak concentration of induction response varies with length of induction in rods (2-day, 3-day and 7-day induction). (J) Frequency histogram of peak Rho-mCherry concentration in live G3U^+^ rods that received Dex treatment for seven days.

Transgenic tadpoles were examined for Dex induction response in whole retina. iXRC1 tadpoles were treated with 10 µM Dex for three days and placed in Dex-free water. After three more days, tadpoles were fixed, retina were cryosectioned and examined using DIC and fluorescence confocal microscopy (Figure 3A). Dex induced the expression of Rho-mCherry in rods and the Rho-mCherry fluorescence was found primarily near the base of the OS (Figure 3A, right panel). In contrast, there was no detectable Rho-mCherry in iXRC1 tadpoles only treated with DMSO for the same period of time (Figure 3A, left panel). Epifluorescence micrographs show G3 translocated into nucleus after induction (*data not shown*). The majority of rods (at least 70%) in transgenic tadpoles responded to Dex induction (*data not shown*). 

To quantify the expression level of the Rho-mCherry reporter, we used high-resolution confocal imaging on live rods [5,9,21,40]. Using constitutive promoters, several features of the Rho-mCherry distribution have been described in *Xenopus* rods that will influence the response profile to Dex (Figure 2C) [5,40]. First, the rhodopsin transgene is synthesized and transported to the OS in a diurnal cycle producing an axial banding pattern with a ~1.5 μm periodicity in animals housed in 12 h dark /12 h light cycles [5]. Second, there is a long-term variation in transgene expression along OS axis with an approximate period of 7-10 days in animals housed in 12 h dark /12 h light cycles [5]. The transgene variation is unsynchronized between cells in the same retina and we call this stochastic variation, since it reflects temporal variation in transgene expression [5]. Thus, we expect a combination of diurnal banding (Figure 2C, middle) and long-term variation (Figure 2C, bottom). For example, in rods, which constitutively express Rho-mCherry under the XOP promoter, periodic axial banding and long-term variation are observed (Figure 3B and 3C). The expression of Rho-mCherry can be observed in both retinal explants (Figure 3B and 3D) and in isolated cells (Figure 3C and 3E), but the latter gives the highest resolution images and were used for all the analysis to follow.

To examine background expression and induction response magnitude, we continuously treated tadpoles with 10 µM Dex for seven days (Figure 2D) and sacrificed the tadpoles for live rod imaging immediately after the treatment. Non-transgenic cells had undetectable fluorescence intensity (Figure 3F and 3G) while cells from transgenic that constitutively expressing Rho-mCherry showed fluorescence throughout the OS (Figure 3B and 3C). Dex treatment did not alter the fluorescence intensity distribution in these two groups. In cells from iXRC1 animals, the fluorescence intensity in the distal (pre-induction) region was undetectable and equivalent to a concentration of Rho-mCherry of <0.2 µM (SD = 0.26, n = 68 rods) which is not significantly different from non-transgenic rods (p = 0.396, t-test). In rods from iXRC1 tadpoles that received 7-day Dex induction, Rho-mCherry fluorescence was only detected at the base of OS where newly synthesized OS membranes are located (Figure 3D, 3E and 3H). Rho-mCherry extended 7-8 µm away from IS/OS junction, close to the distance expected from metabolic labelling studies for OS disk displacement over this period [16,17] (more details see below). It is possible to detect both types of axial variation in these examples (Figure 3 H). 

There was a range in the maximal concentration achieved in these rods (Figure 3I and 3J). The average peak Rho-mCherry concentration was 64.6 µM in rods received 7-day induction (SD=54.4, n=28 rods, Figure 3I and 3J), representing a >300-fold increase of Rho-mCherry concentration after induction. This value represents a significant fold increase over the pre-induction levels. However, since the uninduced Rho-mCherry fluorescence level is below our detection limit, we cannot confidently establish the magnitude of the fold increase; in most cells, it was >100. It is important to note that even though there is a large increase in Rho-mCherry concentration following Dex induction, the levels of Rho-mCherry are significantly lower than the 3 mM endogenous rhodopsin [41]. Rods that were treated for a shorter length of time had lower peak Rho-mCherry concentrations but had less variability (Figure 3I). 

### Reproducibility of induction responses

**Figure 4:**
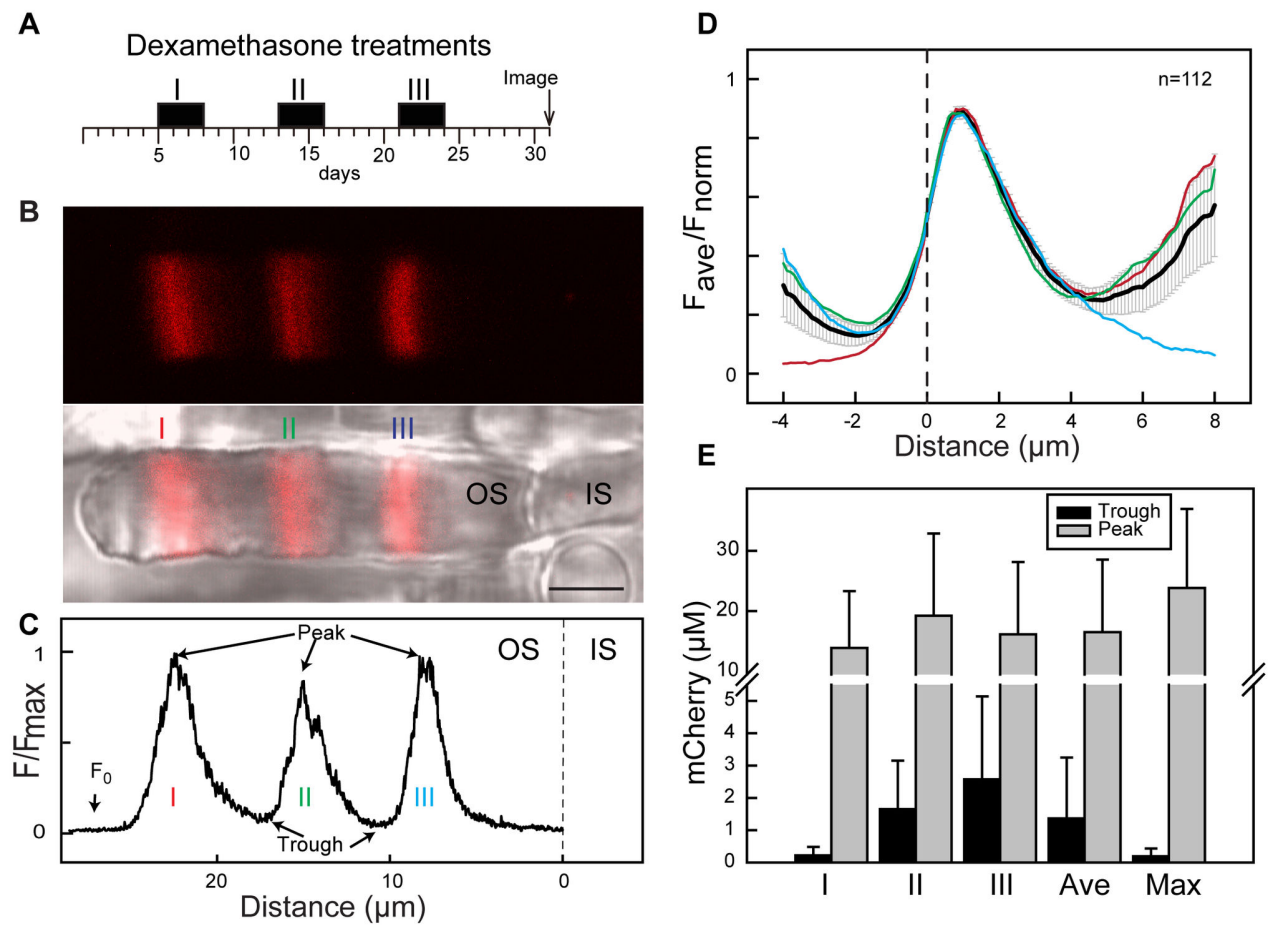
Repetitive induction responses in individual rods. (A) Schematic diagram of the Dex treatment paradigm. (B) Fluorescence (top) and merged with DIC (bottom) images of a live rod that received three Dex treatments. Labels I, II and III indicate fluorescence responses corresponding to the different inductions. Scale bar is 5 μm. (C) Relative fluorescence intensity profile of the rod in (B). For reference, the position of IS/OS junction was set as 0 μm. The maximum intensity (Peak) and minimum intensity (trough) between two induction responses are indicated. F_0_ indicates the pre-induction background expression level. (D) Average normalized fluorescence intensity distribution of rods that received repetitive induction. Data were pooled from 112 inductions of 44 rods whose profiles were extracted from confocal images of 4 tadpoles ranging from St. 52-56. The fluorescence distribution for each rod was aligned at the position where fluorescence in the rising phase is 50% of maximum (designated as 0 μm, dotted line). The average relative fluorescence intensity for all responses is plotted (black line). The average lines of for induction I (red), II (green) and III (blue) are shown. Error bars represent 95% confidence. (E) Average peak and trough Rho-mCherry concentrations derived from the fluorescence intensity for the three different inductions are shown. The 'Ave' is the average concentration of all inductions. The 'Max' is the maximum response in each rod. Error bars represent standard deviation (n = 61, 66, 45, 172, 68 respectively).

To study the reproducibility of Dex responses in iXRC1 rods, we performed 3-day repetitive induction on transgenic tadpoles (Figure 2E). In this experiment, animals were treated with 10 µM Dex for three days and then without Dex for five days, and then repeated twice more. Finally, the tadpoles were placed in water without Dex for seven days and then sacrificed for imaging. In most rods, there were three bands of Rho-mCherry fluorescence appear in the OS (Figure 4B). These Rho-mCherry fluorescent bands reflect the inductions. They were equally separated by non-fluorescent areas. Because OS disk displacement is unidirectional outward [42,43], the outermost fluorescent band represents the first induction. The fluorescence of Rho-mCherry along the axis of OS was measured and profiled with the distance to OS base (defined as OS/IS junction) to examine kinetics and compare maximal intensity of the various induction responses (Figure 4C). We also found rods with fewer Rho-mCherry bands (*see below*) in cells from the same retina. To compare the reproducibility of the induction responses for repetitive treatments, we chose rods that had two to three detectable responses, which comprised approximated 70% of all responding rods. We normalized the Rho-mCherry induction responses to its peak fluorescence intensity and then averaged them (Figure 4D and Figure S1). There were close overlap in all responses during most of the response period (Figure 4D). However, most responses do not reach pre-induction fluorescence levels during the five-day resting period between inductions and it took seven days for the Rho-mCherry levels to return to pre-induction intensities after the last induction. The Rho-mCherry concentration in the troughs between responses (Figure 4C) was 2.1 μM (SD = 2.1, n = 117) but still above F_0_. These experiments show that iXRC1 rods are able to reproducibly respond to Dex treatment over several weeks. 

**Figure 5:**
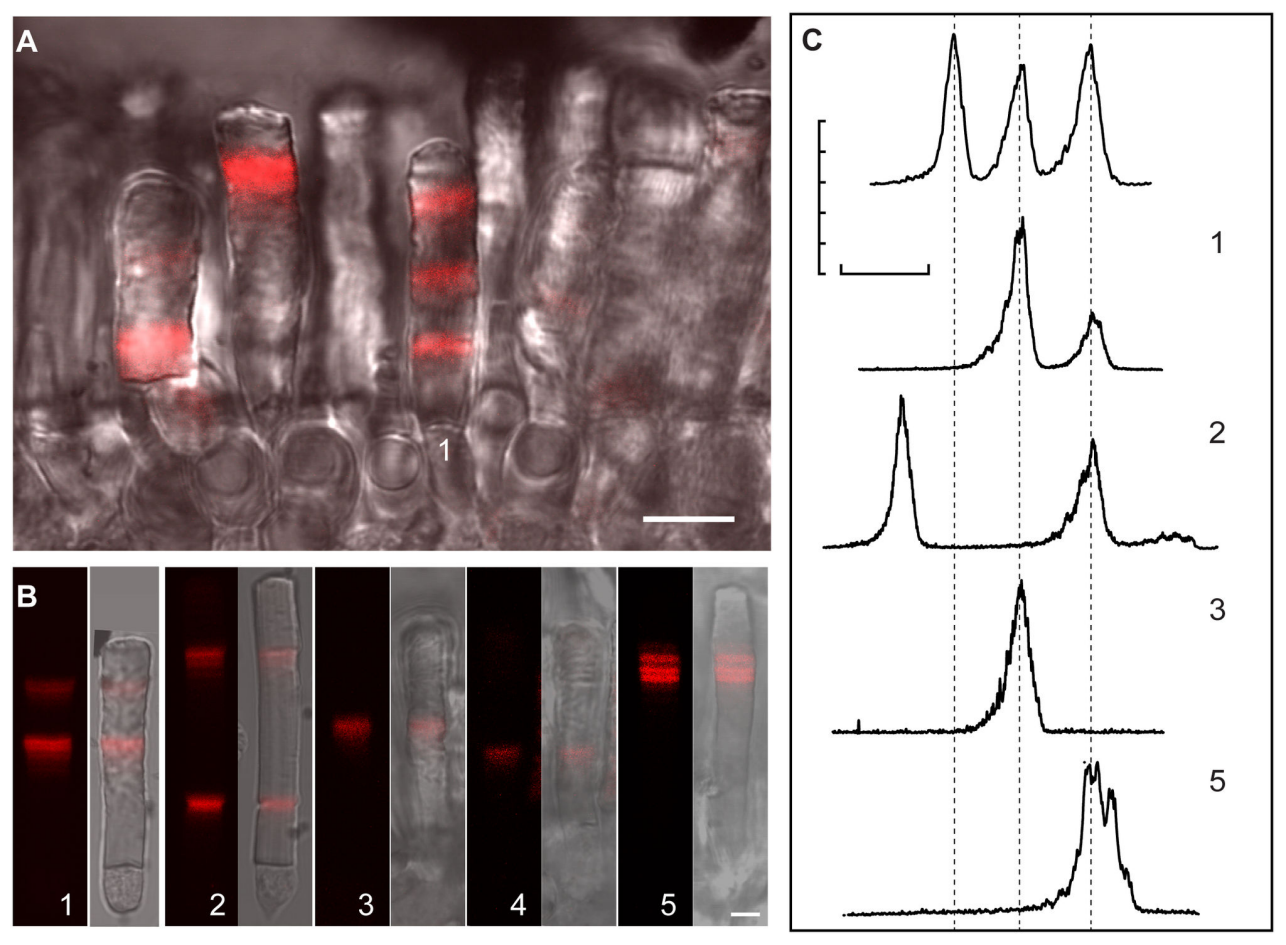
Distribution of Rho-mCherry in live rods after repetitive 3-day induction. (A) Live rods with one to three responses in a retina chip are shown with the fluorescence merge with DIC. Scale bar is 10 μm. (B) Five individual rods with two (2,3) or one (4,5) responses are shown with fluorescence and merged with DIC . Scale bar is 5 μm. (C) Relative mCherry fluorescence intensity profiles of several different live rods, which received same treatment but exhibited different responses. Top scan is from the cell in A with three responses and the others from cells indicated in B. Scale bar on the x-axis represents 10 μm.

We examined the reproducibility of the peak magnitude of the induction between the first, second and third inductions. We measured the fluorescence intensity in the distal part of OS (F_0_) (arrow in Figure 4C) of all rods, which indicates the background expression level of Rho-mCherry before induction, which is <0.22 µM (SD = 0.03, n = 68) (Figure 4E) and is at the same level as in non-transgenic rods. In the first induction, the peak response was 13.9 µM (SD = 9.4, n = 61), representing a 130-fold increase over F_0_ (Figure 4E). The second and third induction responses had Rho-mCherry peak concentrations of 19.2 (SD = 13.6, n = 66) and 16.1 µM (SD = 12.0, n = 45), respectively (Figure 4E). The peak Rho-mCherry concentrations in the three induction responses were significantly above F_0_ (P < 0.001) but not statistically different from each other (one way ANOVA, alpha=0.05). Across all responses, the mean peak response was 23.8 µM (SD = 13.1, n = 68), a 222-fold increase over F_0_ (Figure 4 E). Altogether, the activation and inactivation response profiles of G3U are reproducible in most iXRC1 rods. However, in some rods, there were fewer than three responses (Figure 5A and Figure 5B). The shapes of the responses were not affected, as the remaining responses overlay well (Figure 5C). The most likely reason for the lack of responses to some but not all Dex treatments is the long-term variation in transgene expression, which is stochastic and not correlated between cells in the same retina (Figure 2E). Thus, while the response kinetics and peak magnitude were similar in all responses, iXRC1 rods exhibits transgene variation that in turn can have a significant influence on individual responses. The quantitative impact of the long-term variation in transcriptional activity is considered further in the modelling section.

### Correlation of spatial and temporal induction response profile

**Figure 6:**
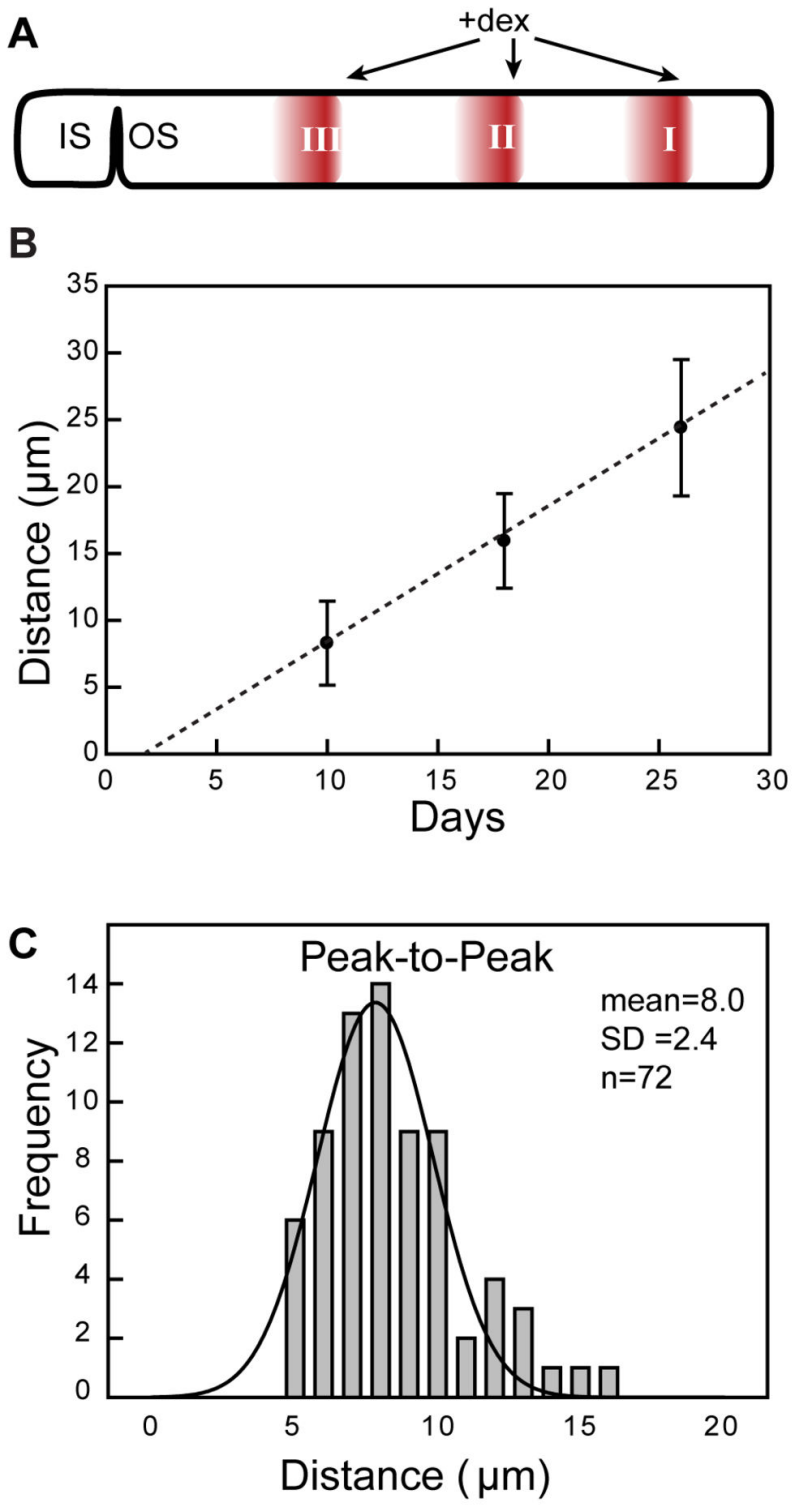
Disk displacement measured from the spatial distribution of induction response peak. (A) Diagram of the Dex treatment paradigm. (B) Correlation of distance of the peak response to the IS/OS junction and time of Dex treatment (Error bar is standard deviation, dash line is the linear regression line. (C) Histogram of peak-to-peak distances in following repetitive inductions with the eight day paradigm. The distance distribution was fit to a Gaussian curve with an R^2^ = 0.96. The mean of peak-peak distance was 8.0 μm (SD = 2.4, n = 72).

We measured the axial position of the Rho-mCherry fluorescent bands in OS of rods following repetitive 3-day inductions (Figure 6A). The average location of the first induction peak is 24 µm (SD = 5.1, n = 44) from the OS base, the second response is 16 µm (SD = 3.5, n = 47) and the third is 8 µm (SD = 3.1, n = 42) (Figure 6B). The first response had a slightly larger variance, which is probably due to OS stretching or swelling during *ex vivo* imaging. Because all rods received the same Dex treatment, we can attribute each Rho-mCherry fluorescent band to a specific induction period and thus estimate the time for the Rho-mCherry band to migrate from OS base to these positions. The fluorescent bands showed a strong linear correlation of distance along the OS and time of induction (R^2^ = 0.99), with a slope of 1.0 µm/day (SE = 0.3) (Figure 6B) and an intercept on the time axis at 1.9 days. There is some uncertainty in this measurement because of the uncertainty in determining accurately the position of response initiation. To better estimate disk movement rates, we also analysed the peak-to-peak distances for sequential responses. The distribution of peak-to-peak distance passed the normal distribution test (Figure 6C) and had an average distance between peaks of 8.0 µm (SD = 2.4, n = 72), which encompassed a period of 8-day (three day treatment with dexamethasone and a five day recovery interval). These results suggest that the daily disk displacement towards the OS tip is 1.0 µm, which matches the estimation of daily OS displacement rates in Stage 54 *Xenopus* raised at 20°C in 12 hours dark/light cycle [13,16,17,22,44-46]. The disk displacement rate among rods is very close, with a standard deviation 0.3 µm/day. 

### Activation phase of the induction response

**Figure 7:**
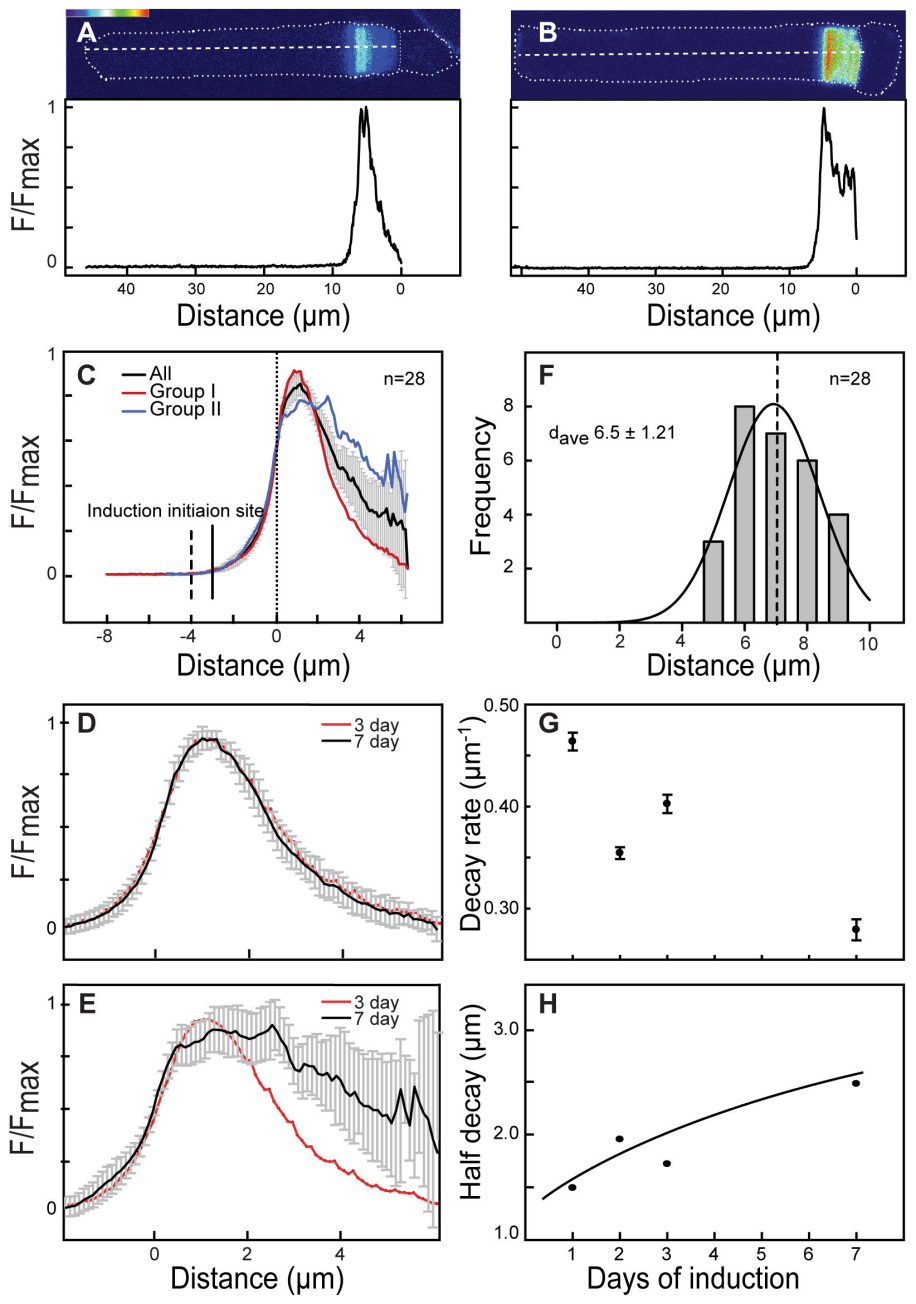
Comparison of activation and inactivation phase of inductions. (A-B) Tadpoles (St. 54) were treated with 10 µM Dex for seven days and then sacrificed immediately before imaging. Individual rods were classified into two groups based upon the shape of the Rho-mCherry fluorescence intensity distributions (see text for details): early terminated responses (Group I, A) and prolonged responses (Group II, B). Heat-maps (top) show the fluorescence intensity distribution of two transgenic rods from each group. The relative fluorescence intensity (F/F_max_) of these two rods is profiled (bottom) from the central z-section along the main axis of their OS (dashed line). (C) Average fluorescence distributions of all (black), Group I (red) and Group II (Blue) rods treated for seven days with Dex. Error bars indicate the 95% confidence level. Dash line indicates expected induction start position; solid line indicates the position where the fluorescence is two standard deviations above pre-induction levels. (D-E) Comparison of average responses from rods treated for seven (black) and three days (red) with Dex. Error bars are 95 % confidence levels. (F). Frequency histogram of the distance from response initiation position to outer segment base. An average of 6.5 μm distance was observed (SEM=0.23, n=28) and fit to a Gaussian curve (R^2^ = 0.97). (G) Decay rate of the induction responses from rods that received various lengths of Dex treatment was estimated by fitting to an exponential. Error bar represents standard error from exponential fit. (H) The time for the induction response to drop to 50% of the peak response (Half-decay) as a function of the length of induction is shown.

We investigated the kinetics of activation by analysing the rising phase of the induction response and response peak position in iXRC1 tadpoles that are treated with Dex for seven days prior to imaging. The induced rods were imaged and analysed for profiling the information of fluorescent intensity and distance (Figure 7 A and B). The fluorescence intensities of different rods were normalized and profiles were aligned. We found two groups of rods, group one with an early termination profile (Figure 7A) and group two with a prolonged response (Figure 7B). Both groups had a similar activation phase (Figure 7C) which was also similar to that observed in 3-day repetitive induction experiments (Figure 7D and 7E). In fact, even rods treated for either one or two days showed the stereotypical activation shape (Figure S3). 

To estimate the time for the induction response to reach its peak, we measured the distance between the initiation of the response and the peak of Rho-mCherry fluorescence intensity (Figure 7C). We defined the initiation site as the point where average Rho-mCherry fluorescence intensity was >2 standard deviations above the background fluorescence (Figure 7C and Figure S2A). In 7-day inductions, we found an average distance of 3.0 µm (SD = 1.0, n = 28) (Figure S2B) while for the first response in rods treated repetitively for 3-days, the average distance to reach peak fluorescence was 3.2 µm (SD = 1.0, n = 33) (Figure S2E). Together, the average distance to reach peak fluorescence in rods was 3.2 µm (SD = 0.96, n = 61). Thus, using the estimate of disk displacement rate 1.0 µm/day, it appears that Rho-mCherry expression takes approximately 3 days to reach the peak concentration following Dex treatment. Thus, there is significant delay in achieving maximal rates of Rho-mCherry incorporation into the OS. This delay may result from a combination of pharmacokinetics of Dex in rods and kinetics of transgene expression (*See Discussion*). Based on the above rate of disk displacement, the first detectable fluorescence in 7-day treated rods should be ~7 µm from the IS/OS junction. The measured distance was less at 6.5 µm from IS/OS junction (SD = 1.2, n = 28) (Figure 7F). The 0.5 µm difference translates to ~0.5 day suggesting this as an estimate for the delay between initiation of Dex treatment and the appearance of detectable Rho-mCherry assembled in OS.

### Inactivation phase of the induction response

We investigated the inactivation kinetics which is the falling phase of the induction response from the peak intensity. As mentioned above, rods that received 7-day induction exhibited highly variable inactivation falling phases. Group I had responses that had returned substantially to baseline levels even though Dex was still present (Figure 7A and C). This group had very similar inactivation phases to 3-day induction rods (Figure 7D). By contrast, Group II had more sustained responses (Figure 7B and C). Nonetheless most responses in this group had reductions in fluorescence intensity after three days (Figure 7E). To estimate the time for the induction response to return to baseline, we measured the distance between the peak of Rho-mCherry in OS to the basal level (less than 10% of peak intensity) (Figure S2A). The 3-day induction rods have an average inactivation phase of 5.5 μm (SD = 1.9, n = 26) (Figure S2F). This result indicates additional 5-7 days after the peak response is required for the rods return to pre-induction status. All average inactivation phases from different induction paradigms could be fit by an exponential decay function (R^2^ > 0.97) (Figures S3 and 7G). The inactivation phase for the 3-day induction has an estimated decay rate of 0.40 μm^-1^. This decay rate suggests that these rods took more than 5.6 days to return to 10% peak fluorescence intensity, which is consistent with above estimations. The decay constant calculated for each induction paradigm (Figure 7H) shows a positive correlation between length of treatment with Dex and the half time for decay. This suggests that there may be some process regulating the recovery of the system that is affected by long-term treatment with Dex.

## Discussion

We have developed an inducible expression system in *Xenopus* and implemented this system for rod-specific expression, characterizing the magnitude and kinetics of induction and recovery in a cell that uniquely records reporter expression levels for weeks. We showed that the G3U system exhibits very tight control, having no detectable expression in the absence of Dex yet exhibiting peak concentrations of membrane protein reporter of >10 µM after induction. Besides the iXRC1 line characterized here, we also found similar induction responses in four other iXRC transgenic lines (*data not shown*). Furthermore, the G3U system allows repetitive inductions with quantitatively similar responses in a majority of cells. 

This inducible system also provides a new approach to study rod OS disk displacement. Here, we induced Rho-mCherry expression, whose history was recorded along the OS axis. We found a displacement rate 1.0 µm for tadpoles at stage 54-56 (raised in 20°C with 12 h light/12 h dark cycle). Traditional methods studied disk displacement using pulse-chase methods with radioactive amino acids to label newly synthesized protein followed by autoradiography [42,43]. Our method allows us to use much higher resolution of microscopy via live imaging. Disk displacement is very sensitive to temperature and varies from 0.65 µm/day at 18 °C to 2.4 µm/day at 28 °C [47]. Our results agree well with those values. However, other membrane proteins cannot be easily studied using radiolabeling methods because of their low abundance and the lack of specificity of the radiolabeling approach. Thus, the G3U system opens the way to extend work testing other genetically altered membrane proteins via induced expression. 

**Figure 8:**
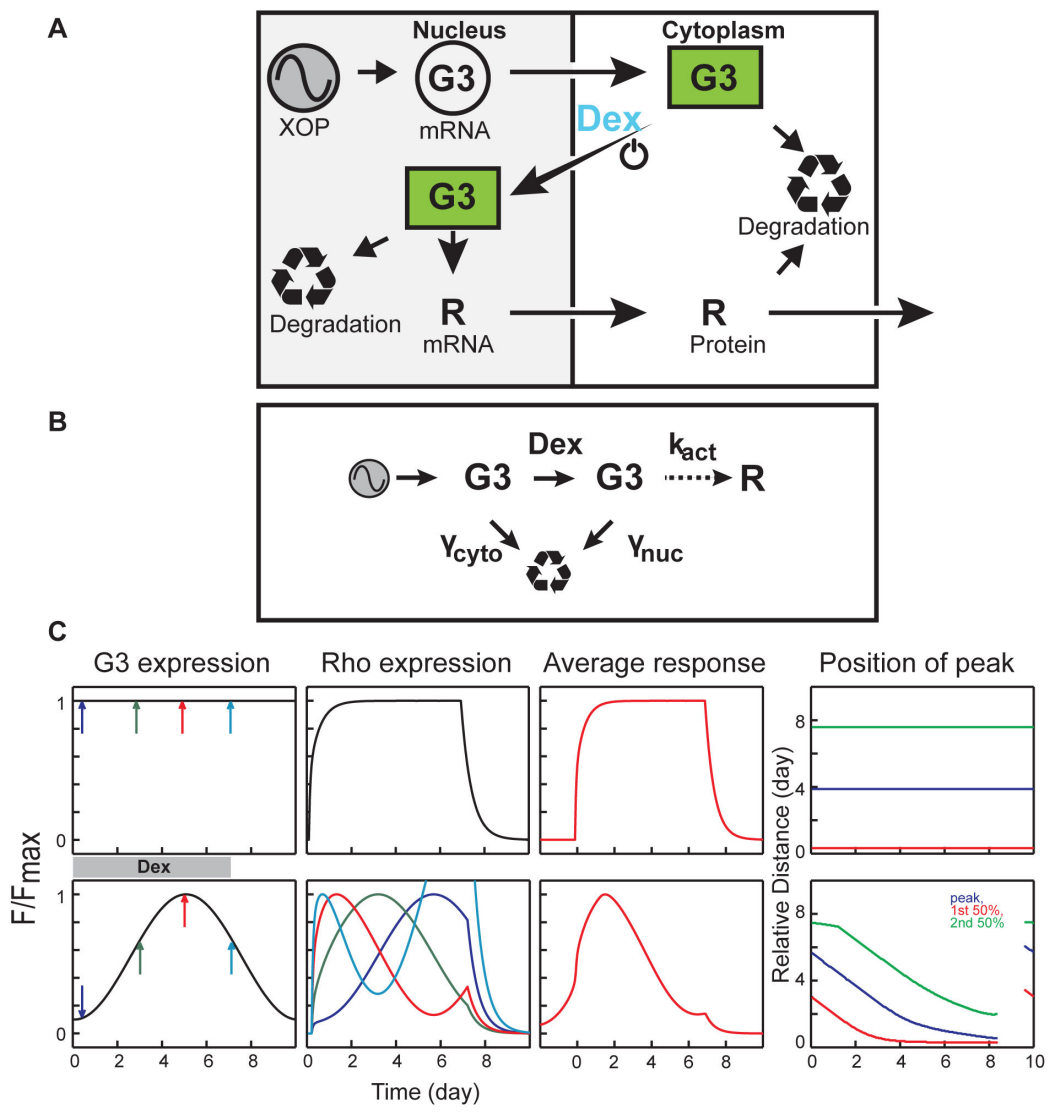
Model to simulate induction response. (A) Schematic of the G3 inducible system. This model includes transcription, translation and transport of Rho-mCherry reporter with both nuclear and cytoplasmic mRNA and protein degradation. (B) Simplified model of inducible system. Transcription and translation steps were combined to yield a model with five parameters: variable sinusoidal synthesis of G3, Dex, k_act_, γ_cyto_ and γ_nuc_ for degradation (See Appendix S1 for details). (C) Simulation of induction responses to seven Dex treatments. Upper panel shows constant G3 expression and lower panel shows variable G3 expression with 10-day period. Color arrows in left-most panel indicate induction at different phases of a 10-day period. The corresponding average induction responses (Rho expression) are plotted in the corresponding color. These responses were obtained by averaging hundreds of simulations responses with randomized phases. The rightmost plots show how the phase of G3 expression changes the characteristic positions in induction response: rising midpoint (red), peak (purple) and falling midpoint (green).

 We used a mathematical model to simulate induction response and to study potential reasons for the variation in the inactivation phase (Figure 8 A). The system produces detectable Rho-mCherry in hours after treatment of animals with dexamethasone. However, it is slower at reaching steady state and recovery than expected, typically taking several days to reach peak synthesis rate and longer to return to baseline after removal of inducer. The reasons for the relative slow rate to reach peak synthesis are unclear. Dex enters the eye equilibrates in the retina within hours in mammals [48] and it has a relatively long half-life (36-54 h) [49] so a steady level of Dex should be reached and maintained relatively rapidly. Although we were not able to use live imaging to determine the rate of transfer of G3 into the rod nucleus, we estimate that the half time for G3 transport is less than 1 hour at 20°C base on the cell culture results (Figure 1B). G3 binds cooperatively to UAS and activates transcription at a concentration as low as 5 μM (Figure 1B) and the rate of Gal4 binding is, ~10 min [50,51]. Rho-mCherry is transported from the Golgi to OS within 1-2 h [20]. Thus, it appears that the concentration of G3 is an important determinant of the induction rate and suggests that the long-term variation in G3 levels could limit the rate of Rho-mCherry production. The long-term variation of transgenic expression in most rods constitutively expressing Rho-mCherry can be fit with a sinusoidal function (Figure S4). Thus, we explored the implications of this type of variation on the induction response using a simple linear model. 

In this model (Figure 8B, Appendix S1), transcription and translation were combined to a single synthesis step to yield a model with only five parameters: variable sinusoidal synthesis of G3, concentration of Dex, rate of synthesis k_act_ and two degradation steps γ_cyto_ and γ_nuc_ for degradation (*Details in Appendix S1*). A random phase in the sinusoidal function was selected to represent Rho-mCherry expression unsynchronized among rods in the same retina [5,52]. Since induction may occur at any point in the varying G3 expression cycle, we expect a variety of Rho-mCherry induction response shapes (Figure 8C). The shapes of induction responses were sensitive to the G3 half-life used (Figure S5). Since we were not able to measure the G3 degradation rate, we used a half-life of four hours based on pulse-chase measurement of Gal4-VP16 degradation rates [53]. This value also generated relatively stable responses in our simulation. Using these parameters, we found that application of Dex during the phase of G3 cycle when concentrations are increasing generates a single peak. Conversely, when induction starts at later points of the cycle where G3 concentrations are falling, several weaker peaks are generated (Figure S5). When we averaged many induction responses that were generated at random phases of the G3 cycle and aligned them at the 50% peak intensity, we found an induction response with fast activation and slow inactivation phase which was similar to the actual induction responses in *Xenopus* rods (Figure 8C represent 7-day induction). The rate of inactivation was relatively insensitive to the length of Dex treatment, as was found in the experimental results. Thus, the stochastic long-term variation in G3 expression could explain the basis for the shape of the induction response.

Many regulatory proteins expressed in the vertebrate eye have functions early during development, cellular differentiation and later maintaining normal function in the adult. Disturbance in the structure or function of the protein may alter both differentiation and cell maintenance. In order to overcome this experimental difficulty for studying transcription factor function in adult rods, a tightly controlled inducible system is needed. The system described here has potential utility for these future studies. 

## Supporting Information

Appendix S1 **Mathematical model of inducible expression.** A simple mathematical model of the inducible expression system in which the G3 levels have a slow sinusoidal temporal variation is presented.
(DOCX)

Figure S1 **Repetitive induction responses in individual rods from each Dex treatment.** The fluorescence distribution for each rod was aligned as described in Figure4. The average relative fluorescence intensity for the indicated number of rods is plotted (*solid line*). The average line of induction I (red), II (green) and III (blue) are listed from top to bottom. Error bars are 95% confidence levels.
(TIF)

Figure S2 **Spatial distribution of induction responses.** (A) Schematic diagram of a rod that was treated for seven days with Dex prior to imaging. The point of Rho-mCherry response initiation (1) and peak intensity (2) are indicated. “Origin” indicates the position of outer segment base. (B). Frequency histogram of the distance from response initiation to peak (1 to 2). The average distance is 3.0 µm (SEM = 0.18, n = 28) and the distribution fits a Gaussian curve (R^2^ =0.99). (C). Frequency histogram of the distance of the response peak to IS/OS junction (2 to Origin). The average distance of response peak to outer segment base is 3.5 µm (SEM = 0.23, n = 28) and the distribution fits a Gaussian curve (R^2^ = 0.98) (D).Schematic diagram of a rod after repetitive 3 days inductions. The position of minimum fluorescence between inductions is indicated (3) and the other labels are the same as in A. (E-F) Average width of rising (E) and falling (F) phases are shown for the different responses. Error bars represent standard deviation.
(TIF)

Figure S3 **Comparison of induction responses in rods treated with Dex for different durations.** Average fluorescence distribution in rods from iXRC1 tadpoles that received 1-day, 2-day, 3-day and 7-day induction. The average line of 3-day induction was drawn in other three plots for comparison (red). Error bars are 95% confidence levels.
(TIF)

Figure S4 **Long-term and diurnal variation in rods constitutively expressing Rho-mCherry .** A rod constitutively expressed Rho-mCherry shows considerable axial variation in the fluorescence intensity distribution (*Upper panel*). The axial fluorescence intensity profile of Rho-mCherry along the axis of OS is shown below (*black line*). The smoothed fluorescence intensity profiles (*red line*) had two components that could be isolated. First the long term variation (*green line*) can be fit with sinusoidal function (black dashed line) and the diurnal variation (*pale green line*) which is less well fit by a sinusoidal function with a shorter period. This rod was from an animal housed at 22 °C and has a faster disk displacement rate than those in the Dex experiments.
(TIF)

Figure S5 **Simulation of induction responses with different G3 half-life.** Simulation of induction responses with 10-day induction and a G3 has a 10-day sinusoidal expression pattern. The G3 expression levels (*red line*) are shown for four unsynchronized hypothetical rods as a function of time. Note that Dex induction (*blue line*) occurs at different phases in the G3 expression cycle. The calculated Rho-mCherry expression level is shown (*black line*) as a function of the G3 degradation rate. The gray indicates a duration of 7 days to aid in comparisons with experimental results.
(TIF)
