# Supplementary material for: An Inducible Expression System to Measure Rhodopsin Transport in Transgenic Xenopus Rod Outer Segments
Source: PLoS One. 2013 Dec 6;8(12):e82629. doi: 10.1371/journal.pone.0082629 (PMC3857830; doi:10.1371/journal.pone.0082629)
Supplement: Appendix S1 — Mathematical model of inducible expression. A simple mathematical model of the inducible expression system in which the G3 levels have a slow sinusoidal temporal variation is presented. (DOCX) [file pone.0082629.s001.docx]

Appendix: mathematical model

The model is based on the simplified scheme as described in discussion (Fig. 8B). Only the G3 input, degradation and transportation was taken into account. All transcription and translation steps were considered as a linear function of G3 transcription factor concentration. Thus, the G3 expression is given by:

(1)

is the minimal expression level of the G3, phase of , is amplitude of G3 expression in a range from 0 to 1 and is the period of long-term variation in expression of G3.

The synthesized G3 is continuously deposited into the cytoplasm of the cell and will leave the cytoplasm via degradation (*γ_cyto_*) and transport induced by Dex; both processes are functions of G3 concentration in cytoplasm, [*G3_cyto_*]. Dex concentration is either 0 or 1, thus the change of G3 concentration with time is given by:

(2)

The change in the amount of free G3 inside nucleus equals the inflow of G3 from cytoplasm and degradation of G3 in the nucleus. The overall expression for the rate of change in Rho-mCherry (R) concentration is determined by constant *k_act_* and the concentration of G3 in nucleus, [*G3_nuc_*]. This constant was estimated by curve fitting of the initial phase of Rho-mCherry inductions. Thus:

(3)

The model was written and simulated in Matlab (Mathworks, Natick, MA). G3 expression was simulated with various phases. Different degradation rates of G3 were iterated to test the stability of the model. Rho relative amount were determined and aligned with 50% peak position and averaged. Therefore, the average responses represent a mixture of hundreds of random phase transgenic cassettes activation.
