# Supplementary figures and images for: An Inducible Expression System to Measure Rhodopsin Transport in Transgenic Xenopus Rod Outer Segments
Source: PLoS One. 2013 Dec 6;8(12):e82629. doi: 10.1371/journal.pone.0082629 (PMC3857830; doi:10.1371/journal.pone.0082629)

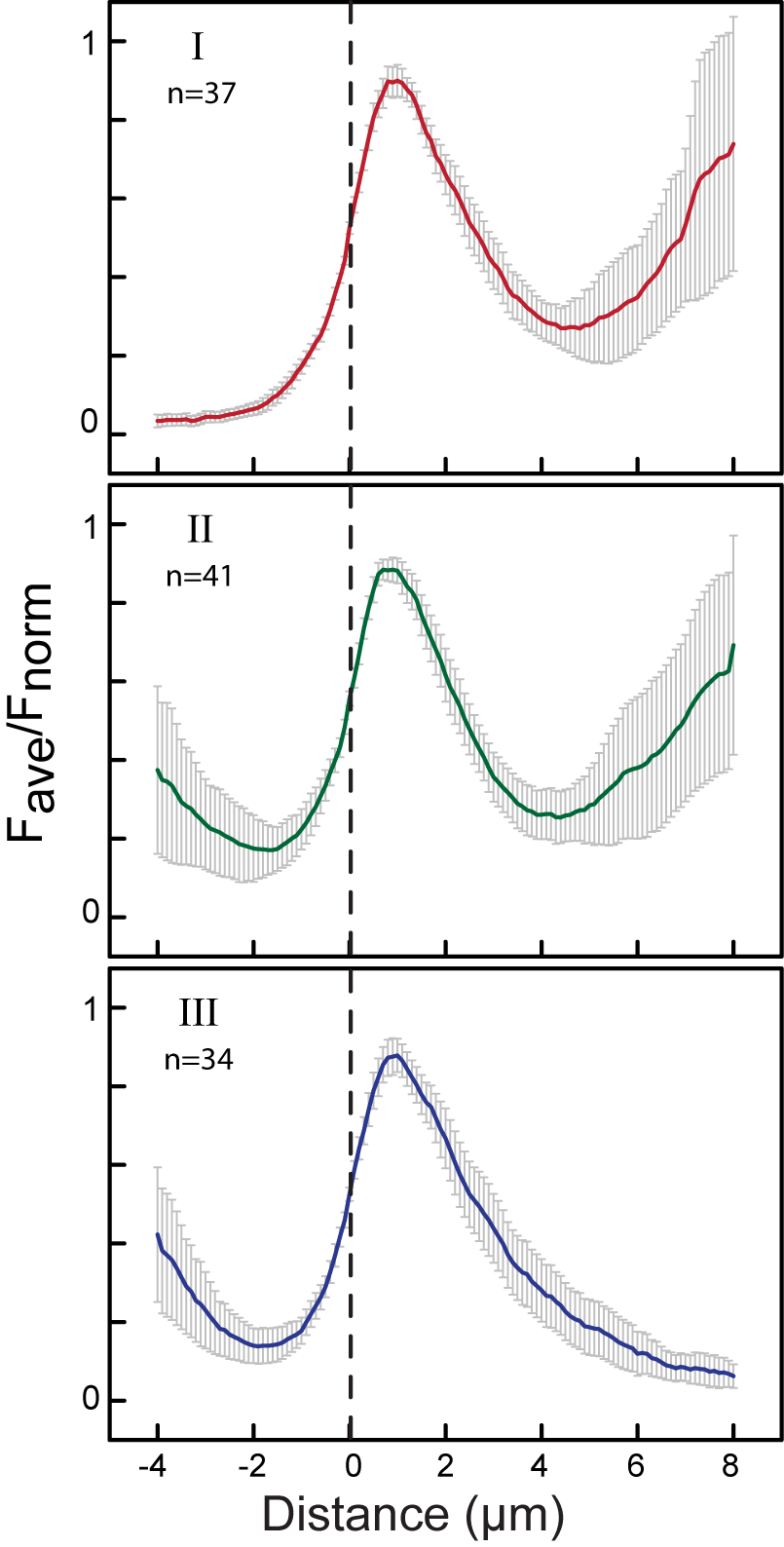

Supplement: Figure S1 — Repetitive induction responses in individual rods from each Dex treatment. The fluorescence distribution for each rod was aligned as described in Figure4. The average relative fluorescence intensity for the indicated number of rods is plotted (solid line). The average line of induction I (red), II (green) and III (blue) are listed from top to bottom. Error bars are 95% confidence levels. (TIF) [file pone.0082629.s002.tif]

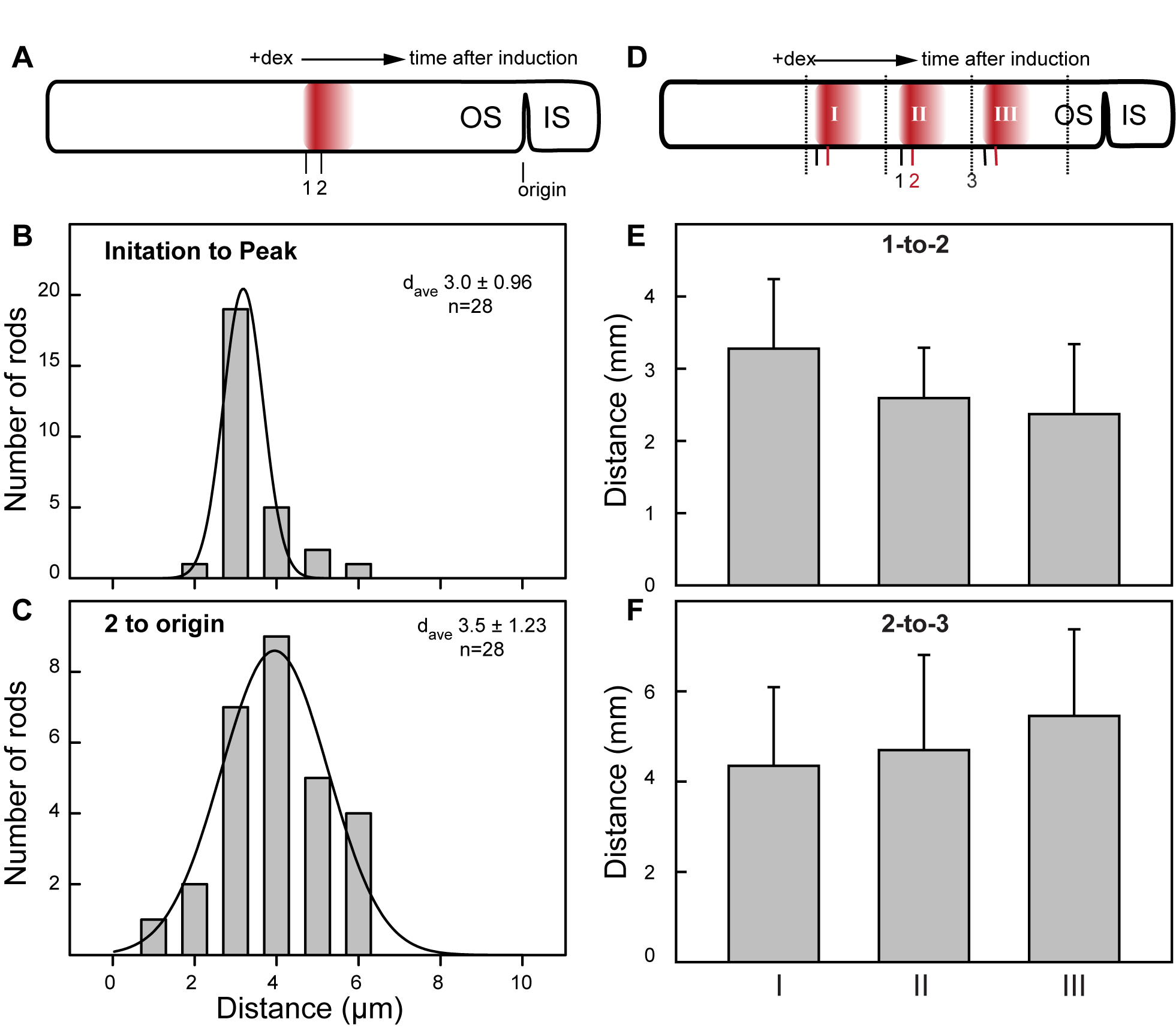

Supplement: Figure S2 — Spatial distribution of induction responses. (A) Schematic diagram of a rod that was treated for seven days with Dex prior to imaging. The point of Rho-mCherry response initiation (1) and peak intensity (2) are indicated. “Origin” indicates the position of outer segment base. (B). Frequency histogram of the distance from response initiation to peak (1 to 2). The average distance is 3.0 µm (SEM = 0.18, n = 28) and the distribution fits a Gaussian curve (R2 =0.99). (C). Frequency histogram of the distance of the response peak to IS/OS junction (2 to Origin). The average distance of response peak to outer segment base is 3.5 µm (SEM = 0.23, n = 28) and the distribution fits a Gaussian curve (R2 = 0.98) (D).Schematic diagram of a rod after repetitive 3 days inductions. The position of minimum fluorescence between inductions is indicated (3) and the other labels are the same as in A. (E-F) Average width of rising (E) and falling (F) phases are shown for the different responses. Error bars represent standard deviation. (TIF) [file pone.0082629.s003.tif]

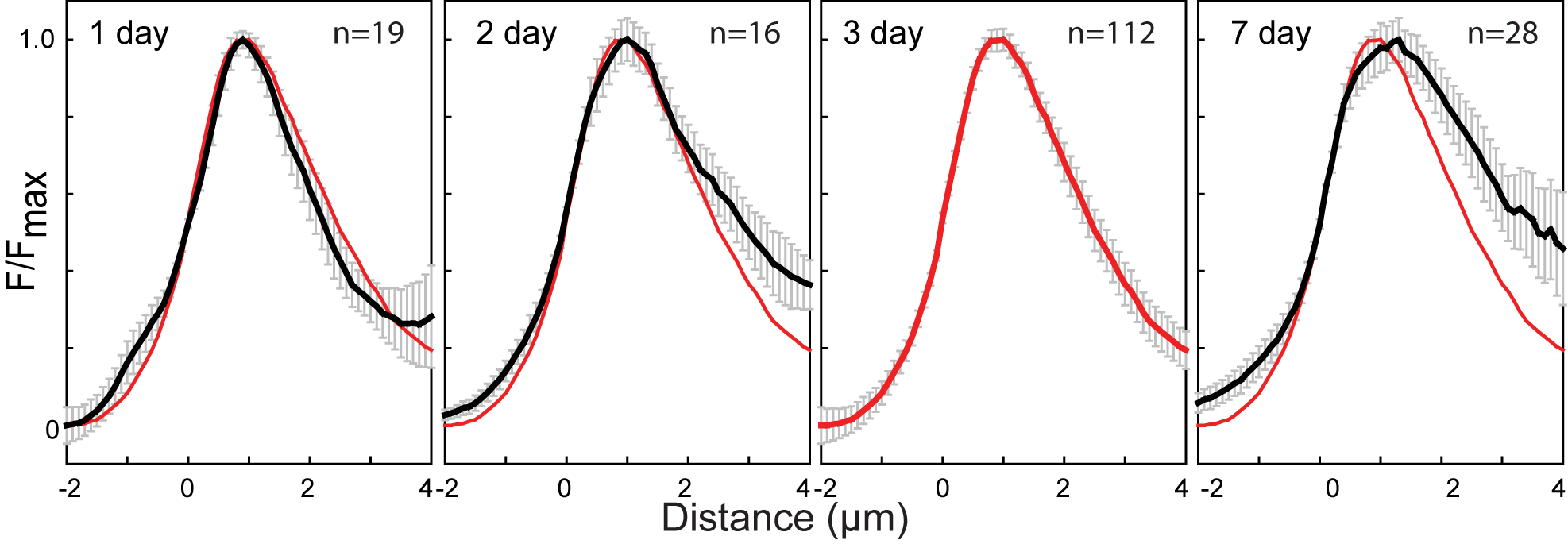

Supplement: Figure S3 — Comparison of induction responses in rods treated with Dex for different durations. Average fluorescence distribution in rods from iXRC1 tadpoles that received 1-day, 2-day, 3-day and 7-day induction. The average line of 3-day induction was drawn in other three plots for comparison (red). Error bars are 95% confidence levels. (TIF) [file pone.0082629.s004.tif]

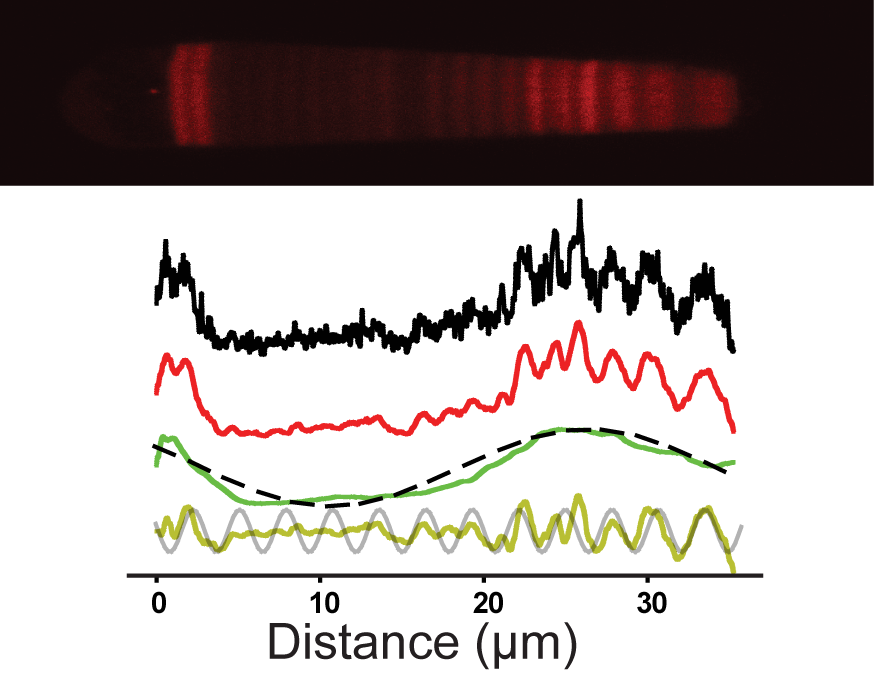

Supplement: Figure S4 — Long-term and diurnal variation in rods constitutively expressing Rho-mCherry . A rod constitutively expressed Rho-mCherry shows considerable axial variation in the fluorescence intensity distribution (Upper panel). The axial fluorescence intensity profile of Rho-mCherry along the axis of OS is shown below (black line). The smoothed fluorescence intensity profiles (red line) had two components that could be isolated. First the long term variation (green line) can be fit with sinusoidal function (black dashed line) and the diurnal variation (pale green line) which is less well fit by a sinusoidal function with a shorter period. This rod was from an animal housed at 22 °C and has a faster disk displacement rate than those in the Dex experiments. (TIF) [file pone.0082629.s005.tif]

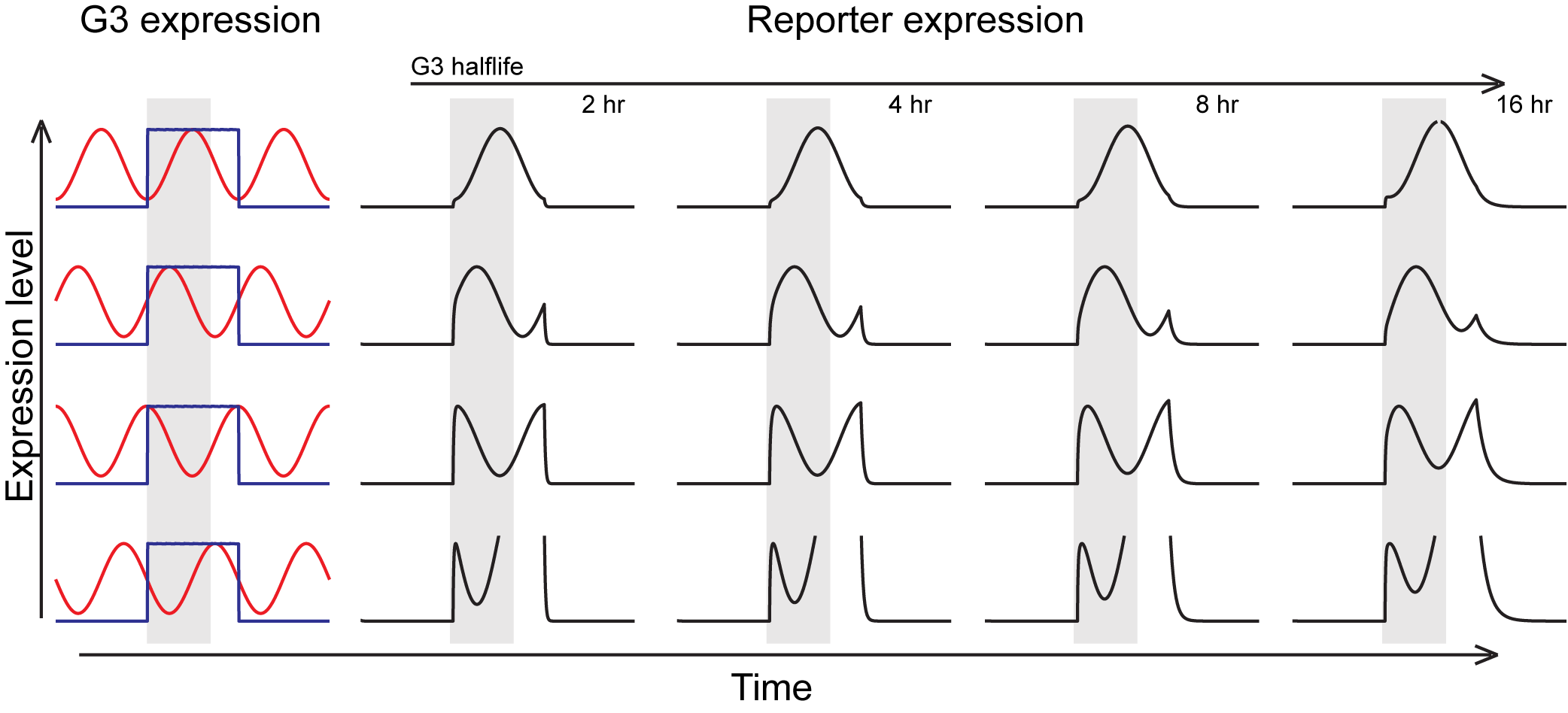

Supplement: Figure S5 — Simulation of induction responses with different G3 half-life. Simulation of induction responses with 10-day induction and a G3 has a 10-day sinusoidal expression pattern. The G3 expression levels (red line) are shown for four unsynchronized hypothetical rods as a function of time. Note that Dex induction (blue line) occurs at different phases in the G3 expression cycle. The calculated Rho-mCherry expression level is shown (black line) as a function of the G3 degradation rate. The gray indicates a duration of 7 days to aid in comparisons with experimental results. (TIF) [file pone.0082629.s006.tif]
